# Effects of Mead Wort Heat Treatment on the Mead Fermentation Process and Antioxidant Activity

**DOI:** 10.3390/molecules22050803

**Published:** 2017-05-14

**Authors:** Sławomir Czabaj, Joanna Kawa-Rygielska, Alicja Z. Kucharska, Jarosław Kliks

**Affiliations:** 1 Department of Fermentation and Cereals Technology, Wroclaw University of Environmental and Life Sciences, Wrocław 51-630, Poland; joanna.kawa-rygielska@upwr.edu.pl; 2 Department of Fruit, Vegetable, and Plant Nutraceutical Technology, Wroclaw University of Environmental and Life Sciences, Wrocław 51-630, Poland; alicja.kucharska@upwr.edu.pl; 3 Lubuski Centre for Innovation and Agricultural Implementation, Kalsk 66-100, Poland; j.kliks@loiiwa.com.pl

**Keywords:** mead, heat treatment, fermentation, antioxidant activity

## Abstract

The effects of mead wort heat treatment on the mead fermentation process and antioxidant activity were tested. The experiment was conducted with the use of two different honeys (multiflorous and honeydew) collected from the Lower Silesia region (Poland). Heat treatment was performed with the use of a traditional technique (gently boiling), the more commonly used pasteurization, and without heat treatment (control). During the experiment fermentation dynamics were monitored using high performance liquid chromatography with refractive index detection (HPLC-RID). Total antioxidant capacity (TAC) and total phenolic content (TPC) were estimated for worts and meads using UV/Vis spectrophotometric analysis. The formation of 5-hydroxymethylfurfural (HMF) was monitored by HPLC analyses. Heat treatment had a great impact on the final antioxidant capacity of meads.

## 1. Introduction

Mead is one of the most ancient drinks known in human history. Processing of bee honey into alcoholic beverages was a tradition of Slavic tribes. The production process involved the dilution of honey and fermentation [1]. There are many types of mead recipes, combining not only honey and water but also a variety of herbs, spices, fresh fruits and juices to create a wide range of products [2]. The ready product contains at least 7% ethanol (*v*/*v*). Polish mead types are classified by volume ratio of mixed honey and water. For example, the mixture in a ratio of 1:1 is called “*dwójniak*” (double mead), and 1:2 is named “*trójniak*” (triple mead), and so on. This classification allows their classification in decreasing order from the most noble with the highest extract “*półtorak*” (1:0.5) and the longest time of fermentation, to meads made with one part of honey and three parts of water—“*czwórniak*”.

The quality of mead is affected by many factors. These include the quality of the raw materials used for production: honey, herbs, juices, spices, fruits, as well as the water. The second factor strongly affecting final quality and overall fermentation performance is heat treatment of the wort. The traditional way of wort preparation is gentle boiling [2] involving the continuous removal of foam from the boiling wort surface. Nowadays, wort pasteurization which is less laborious and more energy efficient is becoming more popular [3]. The third method of wort preparation is the least popular in large-scale mead production, and just involves dissolving honey in the correct volume of water. The lack of heat treatment can cause many problems during fermentation, mainly caused by naturally occurring rich microflora with an abundance of lactic and acetic bacteria, as well as other anaerobic microorganisms [4]. Thermal treatment is the most common way to ensure aseptic conditions in wort prior to its fermentation.

Mead production is challenging due to the high specific gravity of wort and its low buffer capacity, which causes fermentations to last for an extended time. The generation of organic acids such as acetic and succinic acid during fermentation has a strong impact on the fermentation kinetics. The sum of organic acids and dissolved CO_2_ in fermenting wort produced by yeasts often leads to a rapid pH decrease to values below 3.0, which slows the fermentation process, or can even completely stop the fermentation when the pH drops below 2.5. The amount of acid produced and accumulated strongly depends on the yeast strain used for fermentation [2,5]. All these factors make the mead making process very difficult and time-consuming. Typically, the fermentation time of triple mead ranges between 4–8 weeks and strongly depends on honey type, yeast strain and wort preparation technique [6,7].

Bee honey is well known for its high content of biologically active compounds, but most of them are deactivated during excessive heating, which is still a very common process of wort preparation prior to fermentation. Heating inhibits the activity of enzymes, reduces vitamin content, and helps to remove pollen and other protein sources causing cloudiness and making the clarification process less difficult, but also has a positive impact on the fermentation process by stabilizing and sterilizing the wort before pitching the yeast. Meads produced without any heating retain the bioactivity of honey but the process is more demanding. It often results in the production of many off-flavours, leaving a beeswax-like taste, requires extended fermentation time, and makes clarification more difficult [8].

The phenolic content and antioxidant activity of mead greatly depend on the quality and type of honey used for mead production. Therefore, the influence of botanical origin is significant. Thermal processing has a strong impact not only on organoleptic quality and production process, but it can also alter the total phenolics content (TPC) and antioxidant activity. A well-known indicator of bioactivity is total antioxidant capacity (TAC) measured in different in vitro assays [9].

Currently, there are still only a few scientific reports about the technological aspects of mead production including thermal treatment [10,11]. The lack of a sufficient amount of research data gives an opportunity to ask questions and seek answers for a better understanding of the mead making process.

Our research hypothesis was that the mead wort heat treatment affects the fermentation dynamics and outcome, antioxidant activity and HMF content. Two types of honey, multiflorous and honeydew, were used for the preparation of mead worts. Two different strains of commercial distiller’s yeasts were used in the fermentation process. The objective of this study was to evaluate changes in the fermentation kinetics and total antioxidant capacity caused by heat treatment, as well as to test the possibility of applying distiller’s yeast strains (Safspirit Fruit, Safspirit Malt) in mead production.

## 2. Results

### 2.1. General

**Figure 1:**
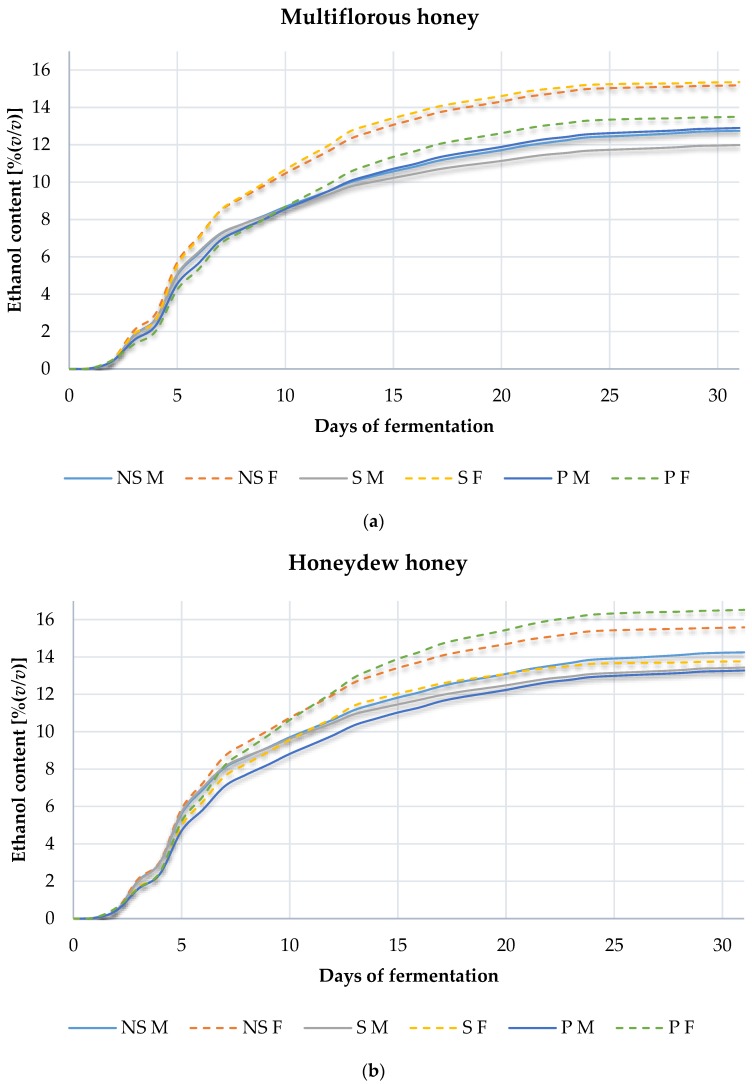
Fermentation dynamics of meads made with the use of multiflorous (**a**) and honeydew honey (**b**). Where respectively: NS—Control, S—Gently boiled, P—Pasteurized, M—Safspirit Malt strain and F—Safspirit Fruit strain.

The fermentation process dynamics were monitored by weighing fermentation flasks and collecting samples for analysis daily. The ethanol production results are shown in the graphs in Figure 1. The highest fermentation rates were observed in samples fermented with the use of the Safspirit Fruit strain. Statistical analysis of data confirmed that observation. The fermentation performance of the Safspirit Fruit yeast strain was significantly (*p* < 0.05) higher than for the other tested strain. Heat treatment and honey type had no significant effect on fermentation dynamics, but there were some clear differences. Analysis of results showed that honeydew honey fermented more vigorously and yielded the highest ethanol concentrations.

**Figure 2:**
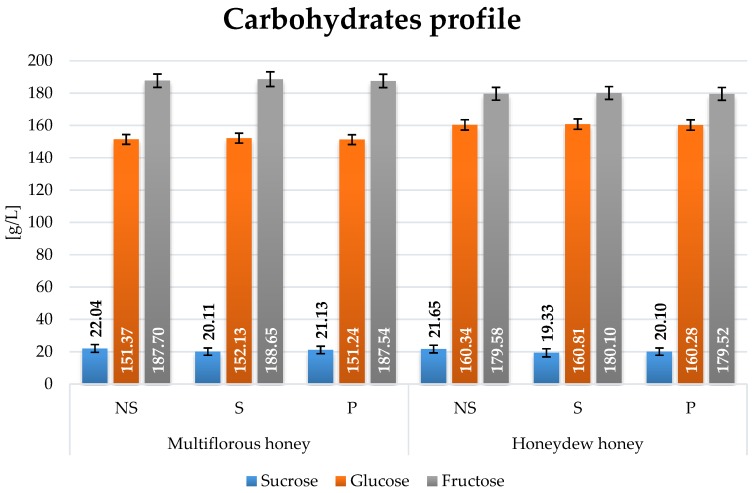
Sugars content in mead wort variants prior to fermentation, where NS—Control, S—Gently boiled and P—Pasteurized, respectively.

Prior to fermentation worts were prepared by the dilution of honey in water setting the total extract of 36 °Bx. HPLC analysis was used to measure concentrations of three major sugars: sucrose, glucose and fructose (Figure 2). Sucrose concentration changed due to heat treatment in comparison to control (NS) samples. Major differences in carbohydrate compositions were found between honeydew and multiflorous honey. To describe the initial composition of sugars in wort the fructose to glucose ratios (F/G) were calculated. The F/G ratio was estimated at 1.24 for multiflorous honey and 1.12 for honeydew honey.

**Figure 3:**
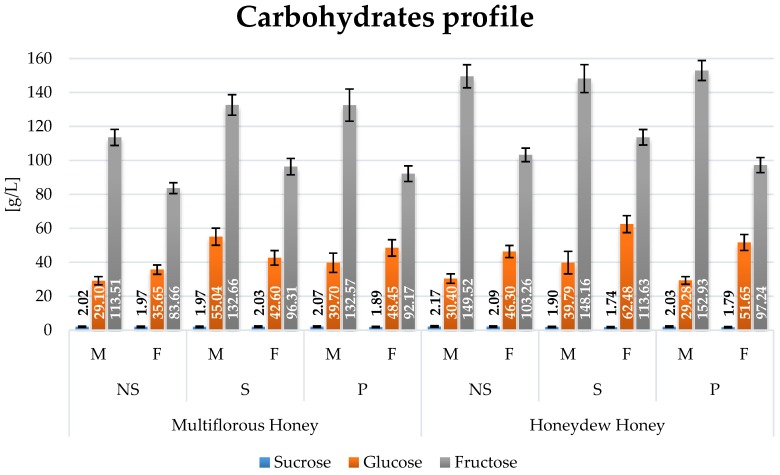
Sugars content in mead variants fermented with Safspirit Malt (M) and Safspirit Fruit (F) yeast strain, where NS—Control, S—Gently boiled and P—Pasteurized, respectively.

The fermentation process reduced the sugar content (Figure 3). About a ten-fold reduction was found for sucrose. The greatest reduction of fructose content (approx. 50%) was found in samples fermented with the use of the Safspirit Fruit yeast strain (*S. bayanus*). The decrease in glucose concentration was also strongly dependent on the yeast strain, and the greatest reduction (approx. 80%) was observed in samples fermented with the Safspirit Malt strain (*S. cerevisiae*).

**Figure 4:**
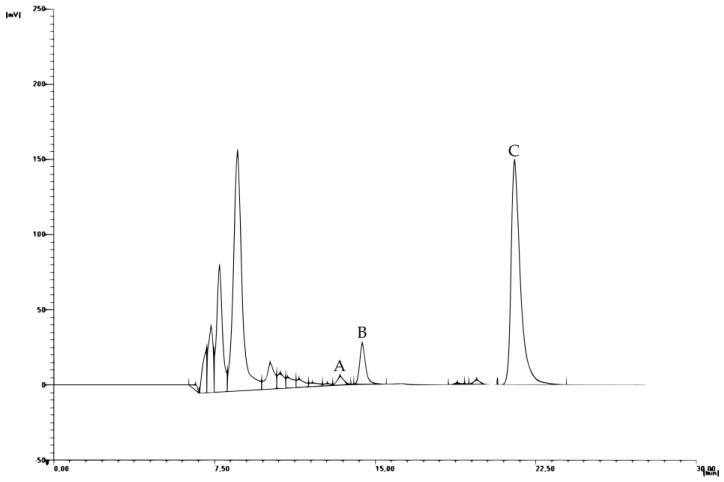
Typical chromatogram of post-fermentation products HPLC analysis, where A—Acetic acid, B—Glycerol and C—Ethanol, respectively.

Analysis of post-fermentation products was performed using high-performance liquid chromatography (Figure 4). Conducted tests were characterized by high repeatability with relative standard deviation (RSD) values below 1% [12].

**Figure 5:**
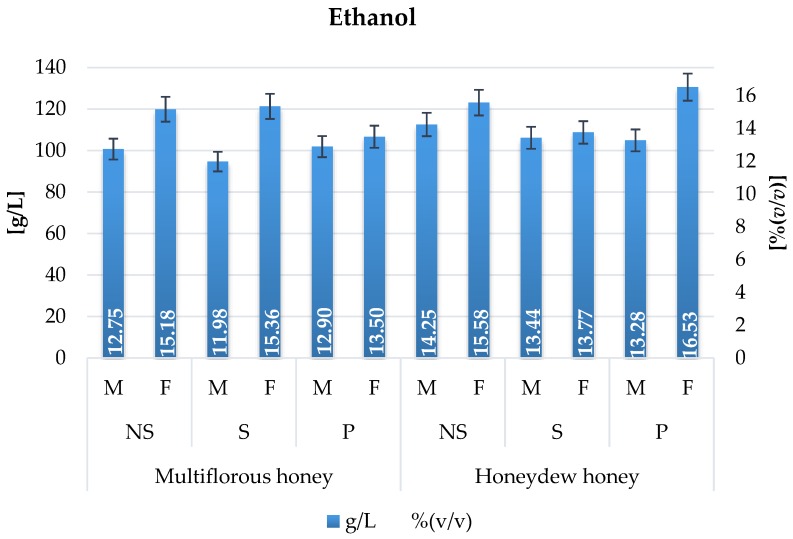
Ethanol content in mead variants fermented with Safspirit Malt (M) and Safspirit Fruit (F) yeast strain, where NS—Control, S—Gently boiled and P—Pasteurized, respectively.

Ethanol content at the end of fermentation ranged from 11.98 to 16.53% vol. (Figure 5), which meets the requirements of the Polish Standard [13] and European Union regulations [14]. Visibly higher ethanol concentrations were observed in worts prepared with the use of honeydew honey. That type of honey is characterized by high conductivity, even reaching over 0.8 mS/cm [15], which is closely related to mineral content and therefore buffer capacity. Buffer capacity is the most common problem in mead production, often sticking the fermentation process and therefore, it slows the overall process and reduces its efficiency.

**Figure 6:**
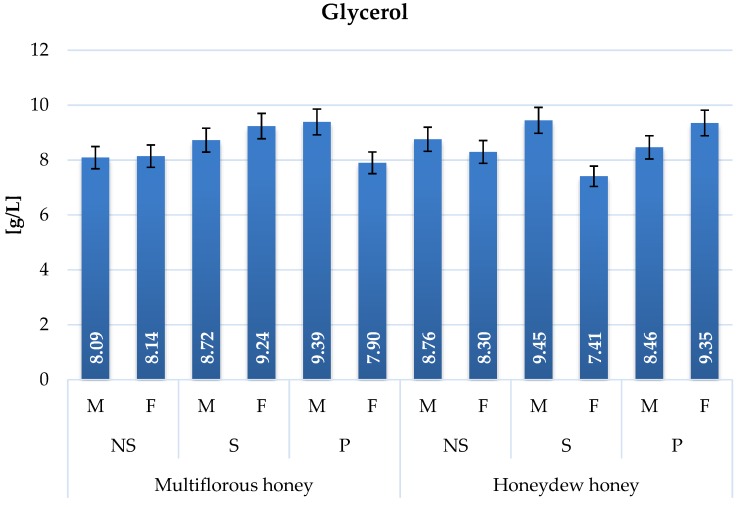
Glycerol content in mead variants fermented with Safspirit Malt (M) and Safspirit Fruit (F) yeast strain, where NS—Control, S—Gently boiled and P—Pasteurized, respectively.

Glycerol concentration ranged from 7.41 to 9.45 g/L (Figure 6). Studies revealed that the production of glycerol by yeasts from *S. cerevisiae* strains accounts for 7–10% of produced ethanol [5]. The high production of glycerol is mainly caused by the harsh fermentation conditions. High osmotic pressure caused by high sugars content leads to the intense synthesis of glycerol as an anti-stress factor. The glycerol output is affected by levels of short chain organic acids, mostly acetic, formic and succinic acids [16]. The inhibitory mechanism of that reaction is related to yeast metabolism and the formation of NADH, which is regulated by acetate abundance. Changes in glycerol concentration strongly correlated with the strain and honey used for wort preparation.

### 2.2. Phenolic Compounds and Antioxidant Activity

**Figure 7:**
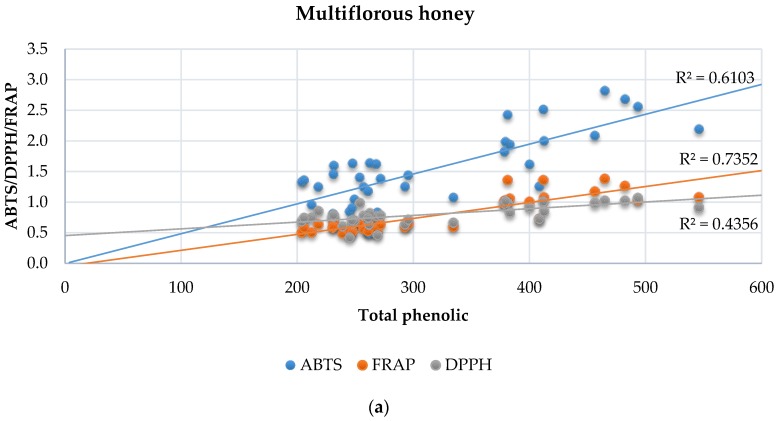

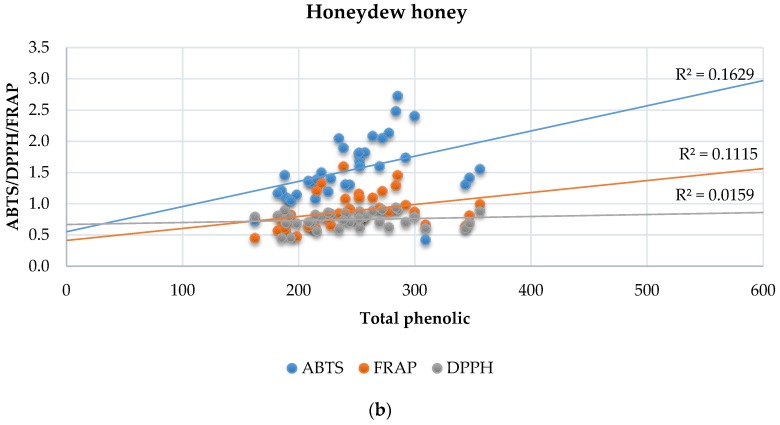
Linear correlation between total antioxidant capacity (TAC) and total phenolic content in meads prepared from multiflorous (**a**) and honeydew honey (**b**).

The comparison of phenolic content and total antioxidant capacity (TAC) measured in three separate in vitro test (ABTS, FRAP and DPPH) is a good indicator of antioxidant ‘quality’. The linear correlations of meads are shown in Figure 7. Total phenolic content in the F-C method and antioxidant activity tests showed strong correlations in meads produced with multiflorous honey. The low coefficient of determination reported for mead samples prepared from honeydew honey was attributed to the higher content of different antioxidants than phenolic compounds, which do not react with F-C reagent, and probably protein-based antioxidants naturally occurring in honey, such as catalase [17].

The analytical data obtained in experiments with the use of F-C reagent showed the highest phenolic compounds content in meads produced using multiflorous honey (Table 1), with an average of 303.02 mg GAE/L in comparison to 236.68 mg GAE/L of honeydew honey-based meads and worts. This was caused by the botanical origin of nectar and pollen collected by bees. Honeydew honey is specific because it is obtained from the secretions of sap-sucking insects or from leaves and other green parts of plants. The highest (247.23 mg GAE/L) initial (control group) total phenolic content was in honeydew honey (Table 1).

The highest phenolic content and antioxidant activities (Table 1) were found in gently boiled mead and wort samples (S). Univariate and two-factor analysis of variance showed a strong positive correlation for wort heat treatment and TAC. Statistical analysis of collected data (Table 1) showed the strong influence of heat treatment on TAC and TPC of mead. The highest antioxidant activities and phenolic content were reported for gently boiled mead samples. The yeast strain used had no significant effect on the TPC and TAC of mead and wort samples. Multiflorous honey meads and wort were characterized by statistically higher TPC, but in TAC there were no statistically significant differences caused by honey type.

### 2.3. Acetic Acid and HMF Content

**Figure 8:**
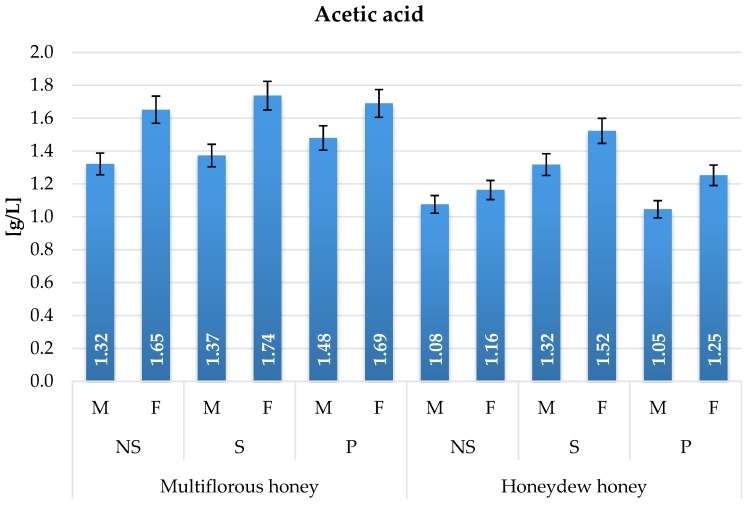
Acetic acid content in mead variants fermented with Safspirit Malt (M) and Safspirit Fruit (F) yeast strain, where NS—Control, S—Gently boiled, P—Pasteurized, respectively.

Acetic acid levels ranged from 1.05 to 1.74 g/L (Figure 8). The highest productivity was observed in samples fermented with the Safspirit Fruit yeast strain. The amounts of acetic acid produced depended on the heat treatment technique and type of honey used for fermentation. Excessive heating during wort preparation in gently boiled samples increased the acetic acid level over the fermentation period in comparison to control samples.

**Figure 9:**
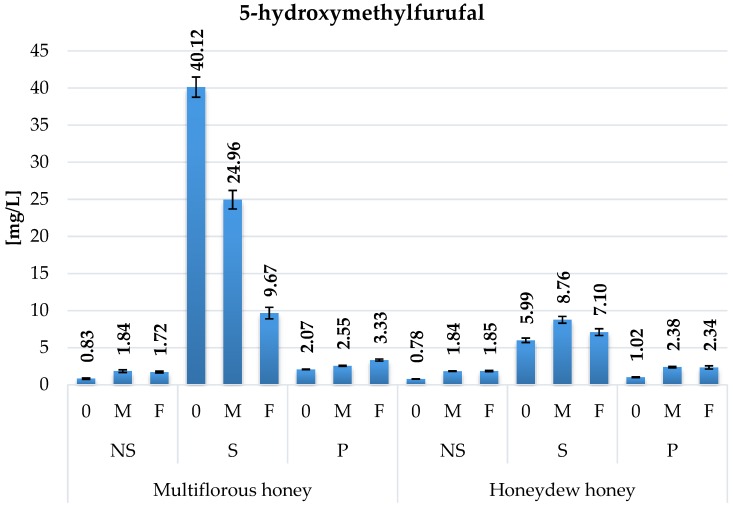
5-hydroxymethylfurfural content in mead variants fermented with Safspirit Malt (M) and Safspirit Fruit (F) yeast strain, where NS—Control, S—Gently boiled, P—Pasteurized, respectively.

Thermal processing significantly affected the 5-hydroxymethylfurfural content in honey worts and meads. The highest growth was reported for gently boiled mead samples (Figure 9). In the great majority of samples an increase of its content during the fermentation was observed, except for the gently boiled meads prepared with multiflorous honey. Fermentation of the above-mentioned worts caused near to 40% reduction of HMF content in samples fermented with Safspirit Malt strain and near to 75% reduction with Safspirit Fruit in comparison to the initial content.

## 3. Discussion

Due to its composition mead has many valuable properties. The ethanol formed during fermentation helps to preserve and extract the phenolic compounds naturally occurring in honey such flavonoids and phenolic acids. Fermentation process had positive impact on TAC and TPC preserving from decrease (Table 1) [18]. Honey is one of the oldest known medicines. It is still commonly used for preparing drugs and is listed in the pharmacopoeia [19].

Nowadays, the trend in food processing is to improve food quality by preservation or even enhancement of its original biological activity [20]. Phenolic compounds, through their antioxidant properties and their wide availability in natural sources with proven efficacy in medicine and cancer prophylactics, are an ideal target for modern research [21,22,23]. It is proven that reasonable and moderate consumption of wine has a positive effect on human health [24], but not all consumers are proponents of red wine and drink white wines only. Recent studies on phenolic compounds content showed higher phenolic content and higher antioxidant activity in meads compared to white wines [11]. Importantly, meads produced in the low-temperature process or without heat treatment retain most of their bioactive properties. Ethanol also facilitates the dissolution and absorption of the phenolic compounds and can have an additional positive influence on the bioavailability of phenolic compounds [25,26].

Acetic acid production during alcoholic fermentation can be affected by many factors, such as carbohydrate source and concentration, pH, level of SO_2_ and nitrogen source, as well as the genetic properties of the yeast strain used [5]. Acetic acid content in mead strongly depends on the initial carbohydrate source and concentration [2,5]. Observed fluctuations of acetic acid content in mead samples were strongly connected with the used yeast strain (Figure 8). Meads prepared with honeydew honey were characterized by significantly lower levels of acetic acid in comparison to multiflorous honey meads (Figure 8). Honeydew honeys are characterized by greater content of glucose (Figure 2), which resulted in lower acetic acid content (Figure 8). Observed increase in heat-treated variants could be caused by the partial evaporation of formic acid naturally occurring in honey during the heating process [2]. It is known that osmotic stress caused by the very high gravity of the fermentation environment slows down the conversion of acetaldehyde to ethanol, which promotes acetic acid synthesis and reduces the ethanol production ratio [5].

Fructose is the precursor of HMF, and the speed of HMF formation is highly dependent on the actual pH of the environment. Because roughly 55% of the sugar in wort is fructose (Figure 2), and during the fermentation a rapid decrease of pH to level 3.0 or below occurs, it produces optimal conditions for its excessive formation [27]. The EU directive [15] regulates the maximum levels of HMF in honey (40 mg per kg for direct consumption or 80mg per kg for the bakery industry), but its concentration in meads is not regulated by any directive. The other important factors which also affect the HMF formation rate in meads are: honey type and quality, heat treatment, and fermentation temperatures [28]. In honey wine production the fermentation temperature should not exceed 25 °C. Higher fermentation temperature promotes the excessive formation of esters which can lead to spoilage, resulting in bad taste and negative off-flavours [29]. 5-hydroxymethylfurfural in higher concentrations is also toxic to yeast [30]. Resistance to HMF strongly depends on yeast strain and genetic properties [31]. Reduction in HMF level was observed in gently boiled samples prepared with multiflorous honey (Figure 9). Yeasts are able to metabolize this substance and reduce its level to safer concentrations. Recent studies on meads available on the market showed high variations in HMF content. Meads produced in lab conditions are far below that limit (Figure 9), with 24.96 mg/L for gently boiled wort fermented with M strain, which reduced its initial content in wort straight after boiling from 40.12 mg/L (by 40%). A study on meads from the Czech market revealed HMF contents in the range of 46 to 280 mg/L [32], which can be attributed to excessive heating during the production or usage of low quality honey [27,33]. In the conducted experiment the concentrations did not exceed 40 mg per litre, with an average of 16.1 mg/L for the gently boiled variant. Another important aspect correlated to HMF content is fact that it is also a strong antioxidant which reacts with F-C reagent and can alter the results (Table 1).

Glycerol is a very important indicator of mead quality. It has a strong influence on the perception of ethanol burning mouthfeel, and in higher concentrations (above 6 g/L) positively affects body perception by increasing the overall viscosity. It was proved that higher concentrations of glycerol positively affect the organoleptic parameters of wines [34]. High yields (7.41–9.39 g/L) are typical for this type of beverage (Figure 6).

## 4. Materials and Methods

### 4.1. Biological Material

Two strains of yeast originally designed for distiller’s worts: *Saccharomyces cerevisiae*-Safspirit Malt (M) and *Saccharomyces bayanus*-Safspirit Fruit (F) from Fermentis (Lesaffre, France) were used in experiments. Dry yeast obtained from producer was rehydrated for 30 min in sterile water before inoculation.

### 4.2. Mead Wort Preparation

Multiflorous honey and honeydew honey were obtained from a local beekeeper from the Lower Silesia region (Poland) and delivered by Miody Polskie Ltd. (Mokra, Poland). The honey was collected in 2015 in an apiary located in the Kłodzko Valley. The received strained honey was subjected to melissopalynological analysis for type determination and used for mead preparation. Honeys were diluted in sterile spring water with mineral content of 284.7 mg/L in a volumetric proportion of 1:2 (honey:water). Specific gravity of 36 °Bx was estimated and topped up with the addition of diammonium phosphate (DAP) as a nutrient (0.4 g/L). The mead wort was divided into three portions for different heat treatment conditions. The first one was gently boiled (S) for 30 min and foam was removed. The second portion was pasteurized (P) at a temperature of 65 °C for 10 min and centrifuged for 20 min at 3030 rcf to remove impurities. After cooling, the specific gravity was controlled and set back to 36 °Bx with distilled water. The third portion was prepared without heat treatment-control group (NS) and was only centrifuged with the same procedure as the second variant. pH was measured and each sample was inoculated with two different yeast strains (0.5 g/L).

### 4.3. Fermentation

Fermentation was performed at room temperature (18–20 °C) and monitored by weighing the loss of CO_2_, and continued till the daily loss of weight was lower than 0.5 g. After that the mead was centrifuged for 20 min at 3030 rcf to separate mead from yeast slurry. Biomass and mead were taken for further analyses.

### 4.4. Analytical Methods

#### 4.4.1. Specific Gravity, Sugars Profile and Ethanol Determination

##### Specific Gravity

Specific gravity and extract were tested with the use of an Anton Paar 6000 densimeter (Graz, Austria) equipped with the beer analyzer DMA module. Both honey wort and mead samples were analyzed to determine density, specific gravity and extract (°Bx).

##### Sugars Profile Determination

Fructose, glucose and sucrose content were determined by means of High Performance Liquid Chromatography (HPLC) [35]. Samples for chromatographic analyses were 3-fold diluted with ultrapure deionized water and filtered through a 0.2 μm nylon membrane filter. Analyses were performed using a Shimadzu Prominence chromatograph (Shimadzu Corp., Tokyo, Japan) equipped with a Luna NH_2_ column (250 mm × 4.6 mm) (Phenomenex, Torrance, CA, USA). Samples were loaded onto a 10 µL injection loop and eluted at 40 °C with 80:20 acetonitrile:water (ACN:H_2_O) mobile phase at a flow rate of 1.2 mL/min. The detection of compounds was performed using Shimadzu RID-20A refractive index detection, at a temperature of 30 °C. Integration and quantification were performed using the LabSolutions software (Shimadzu Corp., Kyoto, Japan).

##### Determination of Post-Fermentation Products

Ethanol, glycerol, acetic acid and lactic acid were tested in HPLC analysis [36]. Samples diluted three times with ultrapure deionized water were centrifuged for 10 min at 9280 rcf and the obtained supernatant was filtered through a 0.2 μm nylon membrane filter. Analyses were performed using a Shimadzu Prominence chromatograph (Shimadzu Corp.) equipped with the Rezex ROA-Organic Acid H+ column (300 mm × 7.8 mm) (Phenomenex). The samples were loaded onto a 20 μL injection loop and eluted at 50 °C with 0.005 M H_2_SO_4_ as a mobile phase at a flow rate of 0.4 mL/min. The compounds were detected using a Shimadzu RID-10A refractive index detector, and the detection temperature was maintained at 50 °C. Integration and quantification of chromatographs were performed using Chromax 10 software (Pol-Lab, Warsaw, Poland).

#### 4.4.2. Phenolic Compound Analysis

##### Determination of Total Polyphenols Content

The total polyphenolic content of the meads was determined using the Folin-Ciocalteu (F-C) colorimetric method [25]. Ten times diluted (1:9, *v*/*v*) mead and wort samples (0.1 mL) and F-C reagent (0.2 mL) were pipetted into cuvettes. After 3 min, 1 mL of a 20% aqueous solution of sodium carbonate (Na_2_CO_3_) and 2 mL of distilled water were added. The absorbance at 765 nm was measured after 1 h, and the results were expressed as mg of gallic acid equivalents (GAE) per 1L of mead. Data were expressed as the mean value for three measurements.

##### Determination of Antioxidant Activity in the Reaction with DPPH (2,2-diphenyl-2-picrylhydrazyl) Radical

The antiradical activity was performed using a DPPH assay [21]. 0.3 mL samples of mead and wort diluted twenty times (1:19, *v*/*v*) in ultrapure water were mixed with 2 mL of 0.04 mmol/L DPPH in methanol and 0.2 mL of H_2_O. After 6 min of incubation at room temperature in a dark place the absorbance was measured with a spectrophotometer at 517 nm using disposable polystyrene cuvettes. A calibration curve was prepared with Trolox solution (0.05 × 10^−1^ mmol/L). The data were expressed as Trolox equivalent (TE) of antioxidant capacity per litre of the mead (TEAC, mmol TE/L). All measurements were performed in triplicate.

##### Determination of Antioxidant Activity in the Reaction with ABTS (2,2’-azino-bis(3-ethylbenzo-thiazoline-6-sulfonic acid)) Cation Radical

The antioxidant activity test using the ABTS assay was performed [37]. 0.06 mL samples of mead and wort diluted twenty times (1:19, *v*/*v*) in ultrapure water were mixed with 3 mL with ABTS solution with measured absorption of 0.700 at a wavelength of 734 nm. After 10 min of reaction the absorption of samples was measured. Each sample was tested in triplicate. The data were expressed as Trolox equivalent of antioxidant capacity per litre of the mead (mmol TE/L).

##### Determination of Antioxidant Activity in the Reaction with FRAP (ferric 2,4,6-tris(2-pyridyl)-1,3,5-triazine [Fe(III)-TPTZ])

The ferric reducing antioxidant power assay (FRAP) is based on the reduction of ferric 2,4,6-tris(2-pyridyl)-1,3,5-triazine [Fe(III)-TPTZ] to the ferrous complex at low pH, followed by spectrophotometric analysis [38]. Briefly, the reagent was prepared by mixing 10 mmol/L TPTZ reagent with 20 mmol/L ferric chloride in acetate buffer (pH 3.6). The quantitative analyses were performed by the external standard method using ferrous sulfate (2 × 10^−1^ mmol/L) as the reference standard and correlating the absorbance (λ 593 nm) with the concentration. 0.3 mL samples of mead and wort diluted twenty times with H_2_O (1:19, *v*/*v*) were mixed in polystyrene cuvettes with 0.7 mL of distilled water and 3mL of ferric complex. The results were calculated and expressed as millimoles of Fe^2+^ per kilogram of the honey. The absorbance was read in disposable polystyrene cuvettes using a spectrophotometer. All measurements were performed in triplicate.

#### 4.4.3. 5-Hydroxymethylfurfural (HMF) Determination using High Performance Liquid Chromatography (HPLC)

The amount of HMF was determined using the HPLC Dionex UltiMate 3000 LC system (Thermo Fisher Scientific, Germering, Germany) equipped with a Cadenza C5-C18 (75 × 4.6 mm, 5 μm) (Imtakt, Portland, OR, USA) column. The compound detection was performed with a UV/Vis array detector. The mobile phase was composed of solvent A (0.1% aq. formic acid, *v*/*v*) and solvent B (100% acetonitrile). The elution protocol was as follows: 0–1 min 5% B in A, 20 min 25% B in A, 21 min 100% B, 26 min 100% B, 27 min 5% B in A. The flow rate of the mobile phase was 1.0 mL/min and the injection volume was 20 μL. The column was operated at 30 °C. HMF was detected at 280 nm. The integration and quantification were performed using the Chromeleon-Chromatography Data System (Thermo Scientific Dionex, Sunnyvale, CA, USA).

#### 4.4.4. Statistics

Mean deviations are shown on graphs. Selected data were processed using Statistica 12.5 software (StatSoft, Tulsa, OK, USA) for calculating, a one-factor analysis of variance (ANOVA) with significance level α = 0.05. Differences between means were tested with the Duncan test (*p* < 0.05).

## 5. Conclusions

The conducted research showed that the use of heat treatment accelerates the fermentation process, maintaining good product characteristics. The best fermentation performance was achieved with the Safspirit Fruit yeast strain, which was caused by better use of fructose by the microorganisms. Heat treatment had a significant effect on the antioxidative properties of meads. The downside of the heating process is the formation of HMF, which can affect product quality. TPC and TAC values were affected by heat treatment, and the best results were found in gently boiled samples. The highest concentrations of ethanol and the lowest acetic acid content were found in meads prepared with honeydew honey, indicating good product quality. Carefully planned heat treatment of honey worts for mead production can improve product quality, increasing its fermentation stability.

## Figures and Tables

**Table 1:** Antioxidant activity determined in separate in vitro tests (ABTS, DPPH, FRAP) and total phenolic content determined in the Folin-Cioalteu (F-C) reaction by spectrophotometric methods in honey wort (0) and meads fermented with Safspirit Malt (M) and Safspirit Fruit (F) yeast strain. Where respectively: NS—Control, S—Gently boiled, P—Pasteurized. Result of statistical analysis of homogeneous groups for parameters: ^1^ Honey type, ^2^ Heat treatment, ^3^ Yeast strain, ^4^ Honey type x Heat treatment, ^5^ Honey type x Yeast strain, ^6^ Heat treatment x Yeast strain. Homogeneous groups are marked as: a, b, c, d, and no significant differences are marked with ●.

Honey type	Heat treatment	Yeast strain	ABTS^●+^								FRAP								DPPH^●^								Total phenolic							
			Mean [mM TE/L]				SD ± [mM TE/L]				Mean [mM TE/L]				SD ± [mM TE/L]				Mean [mM TE/L]				SD ± [mM TE/L]				Mean [mg GAE/L]				SD ± [mg GAE/L]			
**Multiflorus**	NS	0	**1.21**	●	b	●	b	●	b	**0.19**	**0.60**	●	b	●	c	b	c, d	**0.07**	**0.86**	●	b	●	b	**a**	c	**0.10**	**228.00**	**a**	b	●	b	**a**	c	**18.32**
		F	**1.28**	●	b	●	b	●	b	**0.49**	**0.53**	●	b	●	c	**a**	c, d	**0.03**	**0.68**	●	b	●	b	**a**	c	**0.07**	**251.50**	**a**	b	●	b	**a**	c	**12.12**
		M	**1.35**	●	b	●	b	●	b	**0.38**	**0.66**	●	b	●	c	**a, b**	b, c	**0.06**	**0.78**	●	b	●	b	**a**	b, c	**0.03**	**241.47**	**a**	b	●	b	**a**	c	**14.81**
	S	0	**2.59**	●	**a**	●	**a**	●	**a**	**0.17**	**1.37**	●	**a**	●	**a**	b	**a**	**0.01**	**1.01**	●	**a**	●	**a**	**a**	b, c	**0.01**	**419.27**	**a**	**a**	●	**a**	**a**	**a, b**	**34.57**
		F	**2.13**	●	**a**	●	**a**	●	**a**	**0.32**	**1.03**	●	**a**	●	**a**	**a**	**a**	**0.01**	**1.02**	●	**a**	●	**a**	**a**	a	**0.04**	**416.97**	**a**	**a**	●	**a**	**a**	**a**	**53.98**
		M	**2.13**	●	**a**	●	**a**	●	**a**	**0.44**	**1.15**	●	**a**	●	**a**	**a, b**	**a**	**0.11**	**0.98**	●	**a**	●	**a**	**a**	a, b	**0.04**	**446.10**	**a**	**a**	●	**a**	**a**	**a**	**34.35**
	P	0	**1.05**	●	b	●	b	●	b	**0.16**	**0.58**	●	b	●	c	b	b	**0.06**	**0.63**	●	b	●	b	**a**	d	**0.15**	**250.43**	**a**	b	●	b	**a**	c	**5.02**
		F	**1.16**	●	b	●	b	●	b	**0.20**	**0.56**	●	b	●	c	**a**	d	**0.06**	**0.75**	●	b	●	b	**a**	c	**0.03**	**259.87**	**a**	b	●	b	**a**	b	**10.11**
		M	**1.39**	●	b	●	b	●	b	**0.05**	**0.56**	●	b	●	c	**a, b**	c, d	**0.04**	**0.74**	●	b	●	b	**a**	c	**0.04**	**213.57**	**a**	b	●	b	**a**	d	**12.42**
**Honeydew**	NS	0	**1.81**	●	b	●	b	●	b	**0.49**	**0.72**	●	b	●	b	b	c, d	**0.07**	**0.68**	●	b	●	b	b	c	**0.16**	**247.23**	b	b	●	b	b	c	**47.25**
		F	**1.39**	●	b	●	b	●	b	**0.25**	**0.67**	●	b	●	b	b	c, d	**0.05**	**0.85**	●	b	●	b	**a**	c	**0.02**	**231.37**	b	b	●	b	**a**	c	**15.78**
		M	**1.19**	●	b	●	b	●	b	**0.10**	**0.87**	●	b	●	b	b	b, c	**0.15**	**0.84**	●	b	●	b	**a**	b, c	**0.02**	**218.97**	b	b	●	b	b	c	**20.21**
	S	0	**1.93**	●	**a**	●	**a**	●	**a**	**0.24**	**0.86**	●	**a**	●	**a**	b	**a**	**0.01**	**0.62**	●	**a**	●	b	b	b, c	**0.01**	**254.80**	b	**a**	●	b	b	**a, b**	**17.64**
		F	**2.20**	●	**a**	●	**a**	●	**a**	**0.39**	**1.27**	●	**a**	●	**a**	b	c, d	**0.13**	**0.86**	●	**a**	●	b	**a**	**a**	**0.08**	**269.57**	b	**a**	●	b	**a**	**a**	**13.73**
		M	**2.13**	●	**a**	●	**a**	●	**a**	**0.27**	**1.16**	●	**a**	●	**a**	b	**a**	**0.09**	**0.87**	●	**a**	●	b	**a**	**a, b**	**0.07**	**266.40**	b	**a**	●	b	b	**a**	**13.05**
	P	0	**1.45**	●	b	●	b	●	b	**0.35**	**1.21**	●	b	●	b	b	b	**0.32**	**0.59**	●	b	●	b	b	d	**0.12**	**215.67**	b	b	●	b	b	c	**18.45**
		F	**1.43**	●	b	●	b	●	b	**0.10**	**0.82**	●	b	●	b	b	b	**0.14**	**0.73**	●	b	●	b	**a**	c	**0.11**	**248.80**	b	b	●	b	**a**	b	**5.28**
		M	**1.12**	●	b	●	b	●	b	**0.30**	**0.54**	●	b	●	b	b	d	**0.07**	**0.83**	●	b	●	b	**a**	c	**0.04**	**177.30**	b	b	●	b	b	d	**10.95**
